# Retroperitoneal laparoscopic hepatectomy of recurrent hepatocellular carcinoma: case report and literature review

**DOI:** 10.1186/s12876-020-01380-2

**Published:** 2020-08-20

**Authors:** Baifeng Li, Tao Liu, Yijie Zhang, Jialin Zhang

**Affiliations:** 1 Department of Hepatobiliary Surgery, the First Hospital of China Medical University, Shenyang, China; 2 Department of Urology, the First Hospital of China Medical University, Shenyang, China

**Keywords:** Retroperitoneal, Laparoscopic hepatectomy, Hepatocellular carcinoma (HCC), Intraoperative ultrasound (IOUS), Case report

## Abstract

**Background:**

Almost all liver tumours can be removed laparoscopically, but some difficult tumour locations complicate laparoscopic surgery. Recurrent liver tumours often pose great difficulties to laparoscopic surgery due to adhesions caused by previous operations. Referring to laparoscopic adrenalectomy, a retroperitoneal approach is proposed to remove liver tumours near the adrenal gland, which will provide a new method for liver surgery.

**Case presentation:**

Our case involves a patient with recurrent hepatocellular carcinoma (HCC) whose last operation was laparoscopic hepatectomy in our department, with a recurrence of HCC 2 years after the first surgery. In this case, based on preoperative CT and MRI, through a retroperitoneal approach, combined with intraoperative ultrasound (IOUS) localization and indocyanine green (ICG) fluorescence navigation, laparoscopic hepatectomy was successfully performed to precisely resect recurrent hepatocellular carcinoma in segment VII. The patient was discharged on the third day after the operation. The AFP decreased to normal levels on the 28th postoperative day.

**Conclusions:**

Retroperitoneal hepatectomy has the advantages of less trauma, shorter operation times, fewer complications and faster recovery for hepatic tumours near the adrenal gland. Accurate localization of tumours is needed to ensure accurate resection; therefore, IOUS and ICG fluorescence are very important. Liver parenchyma was severed strictly according to fluorescent labelling during hepatectomy, which prevented the deviation of liver parenchyma from the plane and ensured that the margin of hepatectomy was tumour-free. In order to ensure a radical resection of the tumour, it may be necessary to enter the abdominal cavity.

## Background

Hepatocellular carcinoma (HCC) is a common malignancy, and hepatectomy is the first choice for the treatment of HCC. The laparoscopic technique has been widely used for liver surgery, and almost all tumours in any location in the liver can be removed laparoscopically [1–4]. However, some hepatic tumours, such as those near the adrenal gland (located in segment VII), are difficult to access during laparoscopic surgery because of their deep anatomical position and the complexities required for exposure of the surgical field [5]. Recurrent liver tumours often pose great difficulties in laparoscopic surgery due to adhesions caused by previous operations and are an important reason for conversion to open surgery [6, 7]. Therefore, laparoscopic surgery will be more difficult for recurrent liver tumours located in difficult locations. Laparoscopic adrenalectomy, a retroperitoneal approach proposed to remove liver tumours near the adrenal gland [8, 9], will provide a new method for liver surgery.

Retroperitoneal hepatectomy for hepatic tumours near the adrenal gland has the advantages of less trauma, shorter operation times, fewer complications and faster recovery. However, to date, there have been no reports about retroperitoneal laparoscopic hepatectomy for recurrent hepatocellular carcinoma. Our case involves a patient with recurrent HCC 2 years after the first surgery and whose last operation was a laparoscopic hepatectomy in our department. Retroperitoneal laparoscopic resection for recurrent hepatocellular carcinoma has not been reported worldwide.

The retroperitoneal space is very narrow, so incision of the peritoneum should be avoided as much as possible. If the peritoneum is incised, gas will enter the peritoneal cavity, leading to the disappearance of the retroperitoneal artificial pneumoperitoneum, and the intestine may enter the retroperitoneal space, which makes the operation difficult. Because of the limited space for retroperitoneal operation, accurate localization of tumours is needed to ensure accurate resection. Therefore, intraoperative ultrasound (IOUS) is very important. Indocyanine green (ICG) fluorescence navigation was used in this operation. The liver parenchyma was severed precisely, according to fluorescent labelling during hepatectomy. Laparoscopic ultrasound was used throughout the operation to ensure that the operating plane remained at the liver parenchyma and that the margin of hepatectomy was tumour-free.

## Case presentation

### Clinical data

**Fig. 1:**
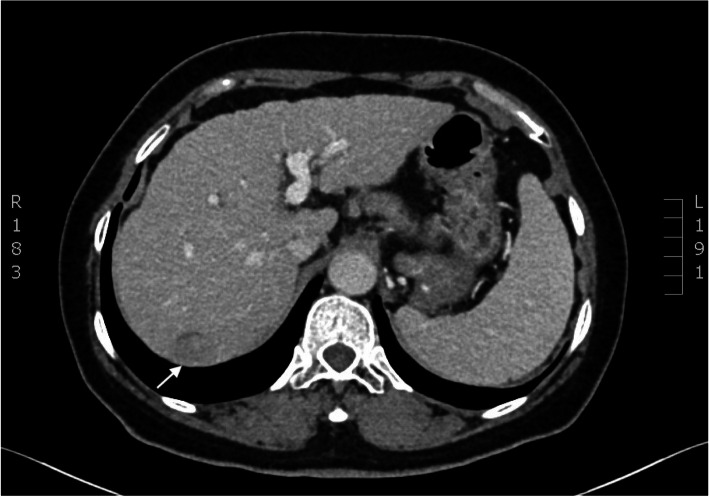
Enhanced CT showed a mass in segment VII of the liver with marked enhancement in the arterial phase and attenuation in the portal and delayed phases, presenting as a “fast in and fast out” pattern

The patient was a 57-year-old male with a height of 165 cm, a weight of 66 kg and a BMI of 24.2 kg/m2. In September 2016, the patient underwent laparoscopic irregular hepatectomy (V and VI liver segments) for primary HCC in our hospital. In July 2018, contrast-enhanced CT scan imaging revealed a neoplasm that showed enhancement in segment VII of the liver (Fig. 1). The laboratory tumour marker AFP was 157.40 ng/ml. Recurrent HCC was diagnosed, and retroperitoneal laparoscopic hepatectomy was performed with the patient’s full knowledge and consent. Seventy-two hours before the operation, indocyanine green was injected through the peripheral vein at 0.25 mg/kg body weight (ICG-r15 = 13.4%).

### Surgical information

**Fig. 2:**
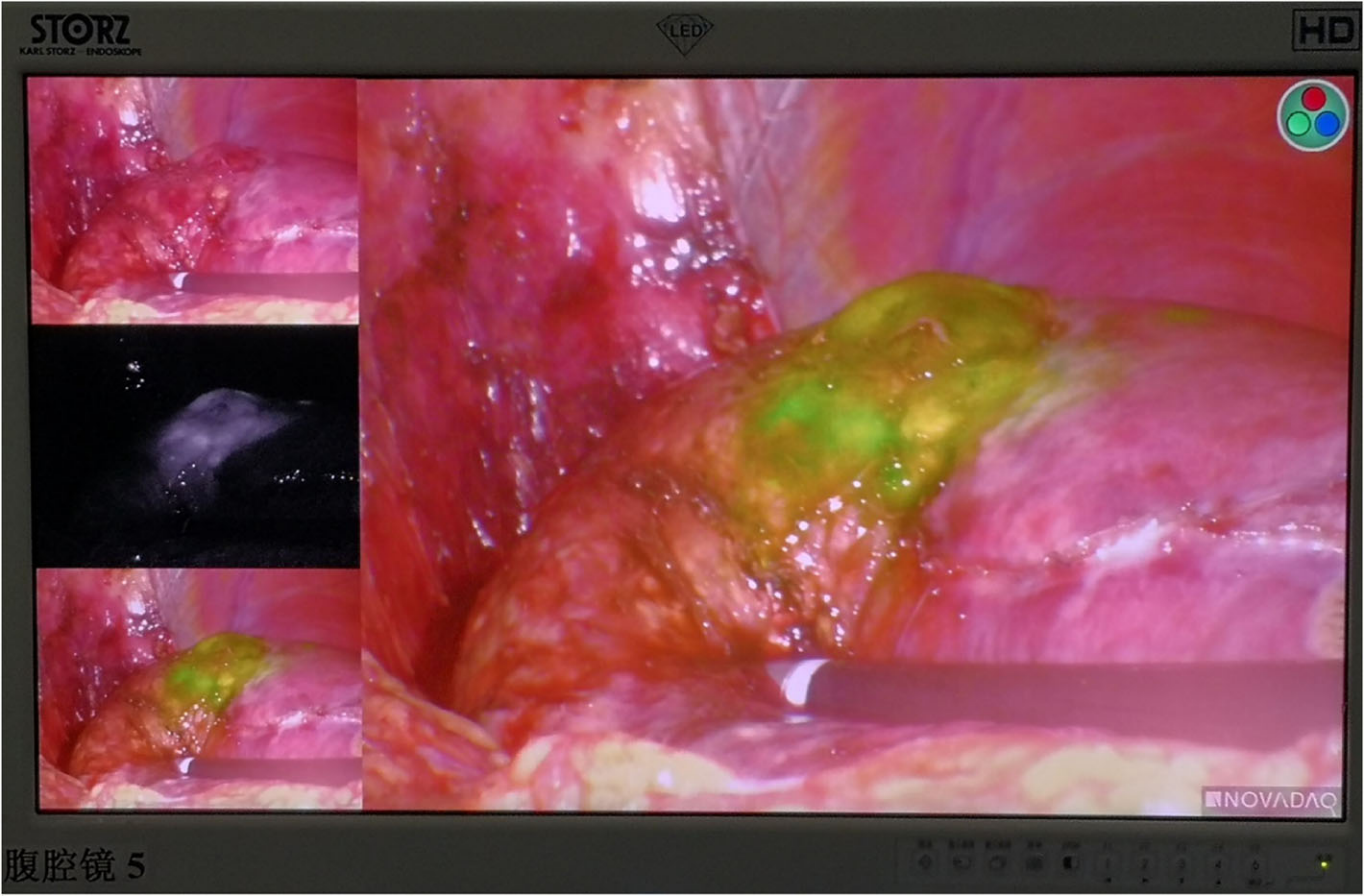
The extent of hepatectomy was determined by ICG fluorescence navigation and IOUS. Under fluorescence laparoscopy, the tumour emitted green fluorescence. The liver parenchyma was severed precisely based on fluorescent labelling and guidance by IOUS to prevent the operating plane from deviating from the liver parenchyma and to ensure that the incision margins were free of tumours

**Fig. 3:**
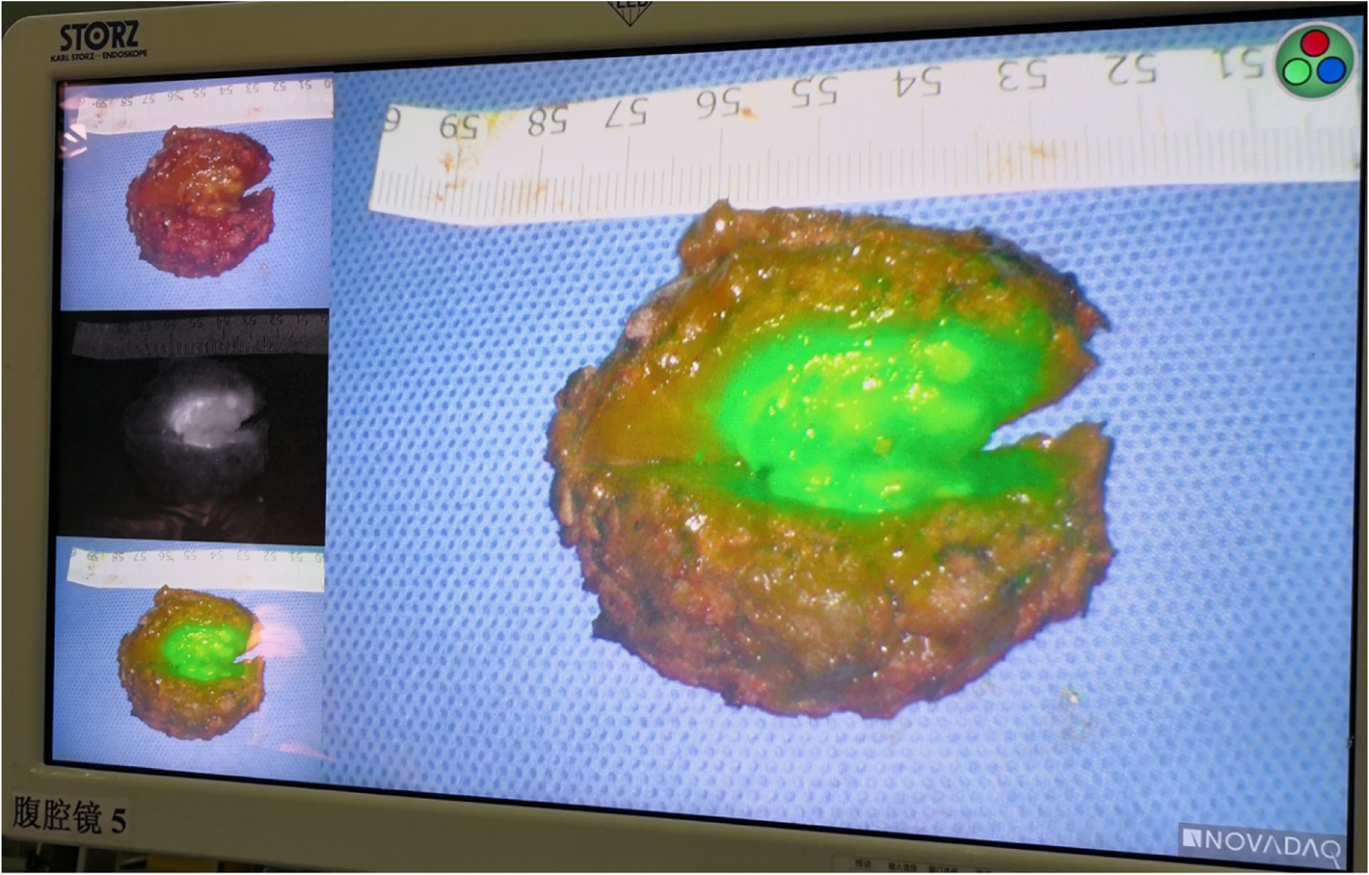
The margin of the incision was more than 1 cm away from the tumour

1. Patient position: Left recumbent position with a high waist cushion.
2. Port site: A 2.0 cm incision was made under the costal margin of the right posterior axillary line, and a finger was inserted into the retroperitoneal cavity for blunt separation. A 1.0 cm incision was made on the iliac crest of the right midaxillary line, and a 0.5 cm incision was made under the front ribs on the right axillary line.
3. Intraoperative procedure: Under fluorescent laparoscopic surveillance, an ultrasound scalpel was used to separate the retroperitoneal space to avoid incision of the peritoneum and to locate the right posterior lobe of the liver. Intraoperative ultrasound (IOUS) was used for localization, and ICG fluorescence was used for navigation. Ultimately, the tumour was completely removed. The margin of incision was more than 1 cm away from the tumour (Figs. 2, 3). During the operation, part of the retroperitoneum was cut into the peritoneal cavity to explore the liver and prevent overlooking other tumours.
4. Operative time: 120 min. Intraoperative blood loss: 20 ml.

### IRB approval was not needed for this study.

#### Microscopic description (Fig. 4)

Light microscopy demonstrated that the tumour cells grew in irregular cords or plates separated by dilated sinusoidal vessels (trabecular pattern) or in focal acinar or pseudoglandular structures. The tumour cells showed cytologic atypia and had abundant pale cytoplasm, large hyperchromatic nuclei, and identifiable nucleoli. Mitotic figures were frequently observed. Dilated bile canaliculi with condensed bile in the lumina were present.

**Fig. 4:**
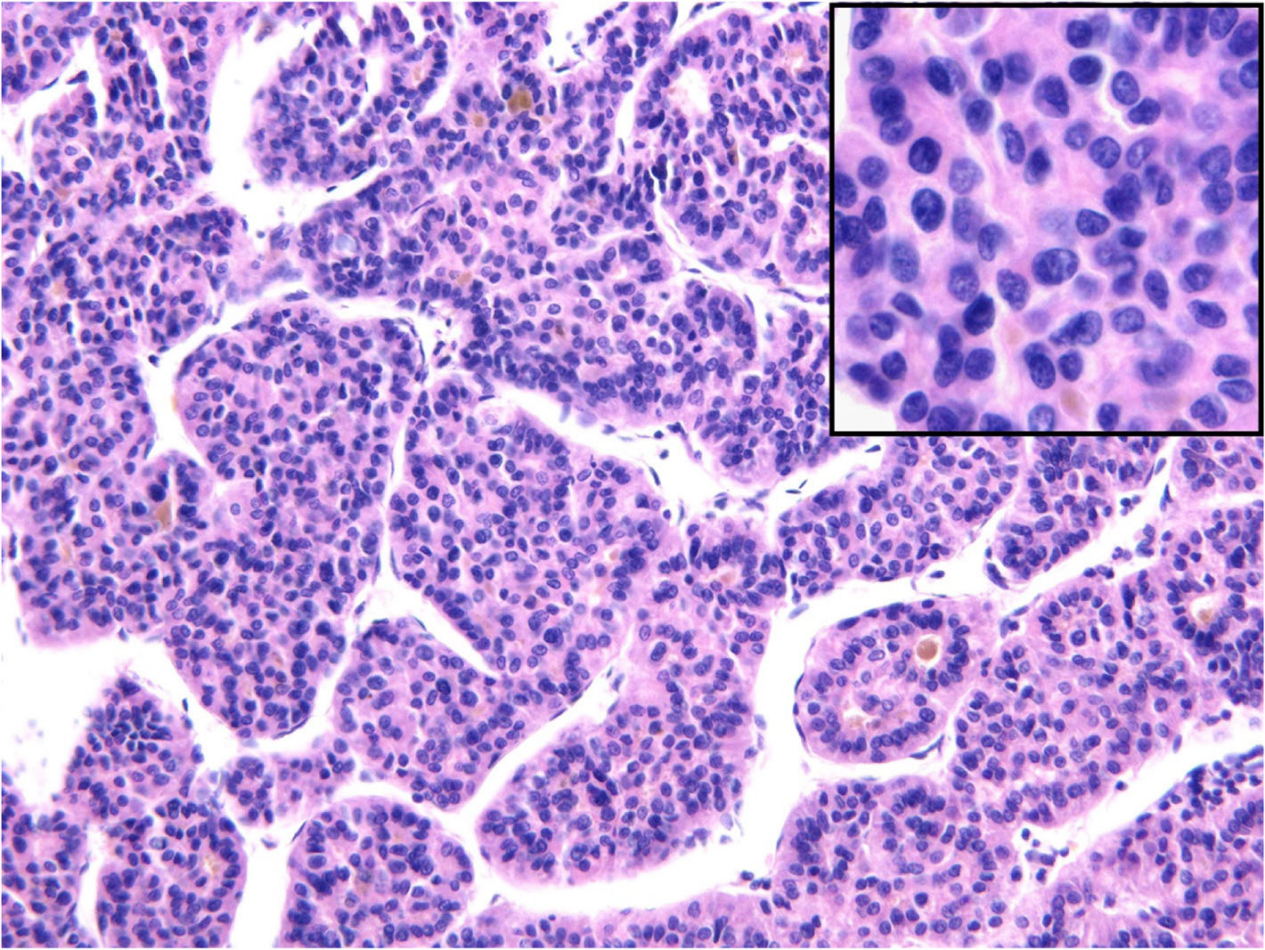
Light microscopy (200×) demonstrated that the tumour cells grew in irregular cords or plates separated by dilated sinusoidal vessels (trabecular pattern) or focally formed acinar or pseudoglandular structures. Dilated bile canaliculi with condensed bile in the lumina were present. The tumour cells showed cytologic atypia and had abundant pale cytoplasm, large hyperchromatic nuclei, and identifiable nucleoli (400× insert)

Immunohistochemical stains: GPC-3 (+), Arginase-1 (−), CEA (−), CK18 (+), CK19 (+), Hepatocyte (+), CK10 (+), CD34 (+), CK7 (−), Ki67 index(60%).

Pathological diagnosis: Moderately differentiated hepatocellular carcinoma.

#### Postoperative follow-up

The patient was discharged on the third day after the operation, and the AFP level decreased to 8.81 ng/ml on the 28th postoperative day. Another contrast-enhanced CT scan was performed, and there was no evidence of tumour recurrence or metastasis. The patient was fully informed, agreed to the treatment plan and was very satisfied with the treatment effect.

## Discussion and conclusions

### Progress and status of laparoscopic hepatectomy

Since Professor Reich [10] first reported laparoscopic hepatectomy for benign liver tumours in 1991, the procedure has undergone development for more than 30 years. The safety and clinical efficacy of laparoscopic hepatectomy have been confirmed, and the indications for use have expanded from benign diseases to malignant tumours. The procedure has been widely used for treatment of hepatic haemangioma, lipoma, focal nodular hyperplasia (FNH), hepatocellular carcinoma and other liver diseases [1, 11].

In 2008, in Louisville, USA [12], and in 2014, in Morioka, Japan [13], experts from all over the world met to formulate a series of specifications for the indications, difficulties and techniques of laparoscopic hepatectomy. It is widely accepted that the best indications for laparoscopic hepatectomy are tumours located in segments II, III, IVb, V and VI of the liver. In recent years, with the improvement of laparoscopic equipment and the accumulation of experience, the size of tumours is no longer a constraint of laparoscopic hepatectomy as long as the location of the tumours allows for a certain operating space under laparoscopy [12]. Laparoscopic hepatectomy is becoming increasingly popular as one of the minimally invasive treatments for hepatocellular carcinoma [13]. Generally, the indications of laparoscopic hepatectomy are almost identical to those of open hepatectomy. As long as a comprehensive preoperative assessment of liver function, basic liver diseases, cirrhosis, portal hypertension, and residual liver volume is made, laparoscopic hepatectomy should not be limited due to the size and location of tumours.

In recent years, laparoscopic hepatectomy has had rapid developments, and laparoscopic left lateral lobectomy has become the standard method [14, 15]. With the improvement of laparoscopic surgery techniques and instruments, the indications for laparoscopic hepatectomy are gradually expanding, and even laparoscopic resection in liver donors can be performed [2, 3, 16, 17]. Compared with traditional open hepatectomy, laparoscopic hepatectomy has the advantages of less trauma, clear operative fields, less intraoperative bleeding, fewer postoperative complications, mild pain, quick recovery, short hospitalization times and decreased mortality. Laparoscopic hepatectomy is suitable for benign and malignant tumours of the liver [1, 15, 18–20] and is also safe and feasible for patients with liver cirrhosis if appropriate cases are selected. In some patients with severe cirrhosis and portal hypertension, the incidence of postoperative liver failure and ascites in laparoscopic hepatectomy is less than that of open surgery [18, 19, 21]. For some patients who are weak, elderly, cirrhotic and obese, the advantages of laparoscopic surgery are more prominent [22, 23]. In addition, during laparoscopic surgery, the traction on the viscera is gentle, and the stress and immune response in patients is less than that in open surgery, which is beneficial for immune function during recovery. Laparoscopic surgery combined with early mobility also decreased the incidence of postoperative reactive pleural effusion and ascites [24].

In laparoscopic surgery, the traditional abdominal incision is replaced by operating ports, which greatly reduces the incision size and the probability of incision complications such as infection, dehiscence and incisional hernia. By using high-definition magnification display equipment, laparoscopic hepatectomy is superior to open surgery for observing and treating the microstructure of liver sections, and the probability of haemorrhage and biliary leakage is reduced. Therefore, laparoscopic hepatectomy is widely used for the treatment of benign and malignant liver diseases [18, 25, 26]. Laparoscopic anatomical hepatectomy has been reported in recent literature for the treatment of primary liver cancer. Not only in perioperative clinical indicators but also in tumour recurrence rate, survival status and other prognostic indicators of long-term postoperative efficacy, the advantages of laparoscopic hepatectomy are increasingly obvious compared with open surgery, which suggests that laparoscopic hepatectomy can be used as a routine operation for the treatment of liver cancer [27].

### Laparoscopic hepatectomy in difficult cases

However, segments I (caudate lobe), IVa, VII and VII of the liver are considered to be difficult sites for laparoscopic surgery because of their deep position, complicated anatomic relationship and unclear surgical field. In the early stage of disease, those segments are regarded as the relative “forbidden zone” of minimally invasive surgery due to the difficulty and high risks [5, 28]. Especially for tumours located in the bare area of the liver, laparoscopic operation is more difficult. The anatomical structure of this area is more complex, and the branches of the hepatic artery, hepatic vein and portal vein are interlaced. Moreover, in patients with hepatocellular carcinoma, there are many vascular networks around the lesion, and the amount of bleeding during operation is large. Therefore, it is easy to lose a clear view of the surgical field. Blind electrocoagulation and clamping after haemorrhage may aggravate tissue damage, which may not only increase the amount of bleeding but also lead to gas embolism. Finally, these complicated procedures force conversion to open surgery and even endanger the life of the patient [29]. Some surgeons have explored new puncture placement, such as the intercostal approach through the chest wall (through intercostal spaces 7–9) and thoracoscopic resection of hepatic tumours in segments VII and VIII through the diaphragm [30]. The advantage of these methods is that they can provide a view of the posterior superior segment of the liver from the top. However, adverse consequences, such as pneumothorax, pulmonary complications and diaphragmatic hernia, which may occur through the thoracic cavity and diaphragm, still need to be carefully considered. Moreover, further multicentre clinical studies are warranted to confirm the safety and long-term oncological benefits of the operation [5, 31].

Based on our experience, laparoscopic hepatectomy for difficult areas of the liver is safe and feasible. However, the location and specific conditions of the tumour must inform the selection of an appropriate surgical position and trocar placement that are conducive to the exposure of the surgical field, thereby reducing the difficulty of resection and improving the safety of the operation. We used the approach of laparoscopic adrenalectomy for reference and performed laparoscopic hepatectomy of segment VII. The patients were placed in the supine position on their left side, with the head higher than the feet. The right retroperitoneal approach was used. The main operating port and observation ports were made at the front, middle and rear axillary lines of the right costal margin to obtain better field exposure and reduce the difficulty of laparoscopic hepatectomy.

It is well known that in patients with a previous history of open hepatectomy or severe cirrhosis, laparoscopic hepatectomy is difficult and has a high conversion rate to open surgery [6, 7]. Most surgeons believe that laparoscopic hepatectomy is more difficult for patients undergoing a second liver surgery [6]. The case described herein involves recurrent hepatocellular carcinoma with severe cirrhosis. During the operation, using the right retroperitoneal approach, the perihepatic ligament is severed, the liver is freed, the right liver is turned forward and downward by gravity so that the operative field is further exposed and the surgery is successfully performed. Laparoscopic resection of recurrent hepatocellular carcinoma via the retroperitoneal space has not been reported before. Of course, this case involves a small hepatocellular carcinoma, which is one of the important reasons for the success of the surgery. If the tumour is more than 10 cm or the lesion is protruding onto the liver surface, which occupies a large amount of space under the pneumoperitoneum, it is difficult for the surgeon to locate enough space to perform the procedure. Hence, these cases are not suitable for laparoscopic hepatectomy [32].

### Application of intraoperative ultrasound (IOUS) and indocyanine green (ICG)

The application of intraoperative ultrasound (IOUS) and indocyanine green (ICG) fluorescence navigation in laparoscopic surgery is also an important factor in the success of this case. Although the development of laparoscopic technology has overcome many of the shortcomings of laparotomy, the surgeon cannot directly contact important organs or structures, and the lack of two-dimensional visual information results in higher risks of laparoscopic surgery than open surgery, which may lead to complications and even death of the patient after surgery [33, 34]. IOUS is a widely used imaging method in clinical settings and has the advantages of convenient operation, high resolution, repeatability and non-invasiveness. The application of IOUS in laparoscopic hepatectomy can overcome the above shortcomings to a certain extent [35]. IOUS can clearly show the diameter and flow of intrahepatic vessels, improve the accuracy of the operation and contribute to the success of laparoscopic hepatectomy [36].

Although IOUS can perform intraoperative tumour identification, it cannot provide real-time and continuous visualization of liver tumours. ICG fluorescence navigation is a real-time and continuous visualization technology that is safe and simple but still has great room for development [37]. With preoperative ICG intravenous injection, fluorescence imaging can be accurate to 2 mm for hepatocellular carcinoma (HCC) and 1.5 mm for hepatic metastases [37, 38]. The role of ICG fluorescent navigation is particularly prominent in laparoscopic hepatectomy because of the loss of tactile feedback and the difficulty of detecting small superficial tumours of the liver [39]. Intraoperative ICG fluorescence navigation combined with IOUS, preoperative CT, MRI, 3D reconstruction and intraoperative frozen pathology can significantly improve the detection and resection of liver lesions.

## Conclusions

In this case, based on preoperative CT and MRI, through a retroperitoneal approach combined with IOUS localization and ICG fluorescence navigation, laparoscopic hepatectomy was successfully performed to precisely resect recurrent hepatocellular carcinoma in segment VII. This kind of case has not been reported in the literature before and may provide a new surgical option for laparoscopic hepatectomy.

## Data Availability

Not applicable.

## Abbreviations

AFP: Alpha fetoprotein
FNH: Focal nodular hyperplasia
HCC: Hepatocellular carcinoma
ICG: Indocyanine green
IOUS: Intraoperative ultrasound
